# Effects of Increased Calcium, Magnesium, and Potassium Ion Concentrations on Survival Conditions, Growth Performance, and Physiological Parameters in Sea Urchin (*Strongylocentrotus intermedius*)

**DOI:** 10.3390/biology14081046

**Published:** 2025-08-14

**Authors:** Xuechun Jiang, Fanjiang Ou, Tongshan Jia, Hao Guo, Peng Liu, Wenzhuo Tian, Shuaichen Wu, Siyuan Chen, Wenping Feng, Weijie Zhang

**Affiliations:** 1 Key Laboratory of Mariculture & Stock Enhancement in North China’s Sea, Ministry of Agriculture, Dalian Ocean University, Dalian 116023, China; 15395747873@163.com (X.J.);; 2 Fisheries College, Zhejiang Ocean University, Zhoushan 316022, China

**Keywords:** *Strongylocentrotus intermedius*, calcium ion, magnesium ion, potassium ion, survival, growth

## Abstract

This study examined the effects of calcium, magnesium, and potassium ion concentrations on *Strongylocentrotus intermedius* through seven experimental rearing groups: two calcium ion levels (550 mg/L and 733 mg/L), two magnesium ion levels (1727 mg/L and 2302 mg/L), two potassium ion levels (533 mg/L and 710 mg/L), and a control. After 45 days of cultivation, 733 mg/L of calcium ions significantly reduced the *S. intermedius* survival rate, while 550 mg/L of calcium ions had no significant adverse effects on survival, growth (SGR), the feed conversion ratio (FCR), gonad colour, or immune enzyme activity, and it significantly increased the gonad index (GI). Meanwhile, 2302 mg/L of magnesium ions caused 100% mortality within 2 days, and 1727 mg/L of magnesium ions significantly reduced the survival rate and SGR while increasing the FCR. Additionally, 710 mg/L of potassium ions had no significant impact on the survival rate but significantly reduced SGR, the GI, and gonad colour while increasing the FCR, whereas 533 mg/L of potassium ions showed no significant adverse effects on survival, SGR, the FCR, gonad colour, digestive enzyme activity, or immune enzyme activity. The study results indicate that when formulating artificial seawater using source water with elevated calcium, magnesium, and potassium ion concentrations for *S. intermedius* aquaculture, calcium ions under 550 mg/L and potassium ions under 533 mg/L are biologically acceptable. However, magnesium ions require adjustment to a narrow optimal range to ensure survival and physiological performance.

## Simple Summary

This study investigated how varying levels of calcium, magnesium, and potassium ions in seawater affect the health and growth of *Strongylocentrotus intermedius*. High calcium ion concentrations negatively impacted survival, while moderate levels supported gonad development without harming growth or immunity. Extremely high magnesium ion levels proved lethal, and even slightly elevated levels reduced survival and growth efficiency. Potassium ions at higher levels impaired growth and gonad quality, whereas lower concentrations had no adverse effects. The findings highlight the importance of carefully balancing these ions in aquaculture, particularly magnesium ions, which must be tightly controlled to ensure sea urchin survival and optimal physiological performance. These insights can guide seawater formulation for sustainable sea urchin farming.

## 1. Introduction

The sea urchin *Strongylocentrotus intermedius* has gradually become the predominant cultivated species in the aquaculture industry in China since its initial introduction in 1989 [1]. Its gonads are globally consumed for their exquisite flavour and rich nutritional value [2]. This species exclusively thrives in high-salinity marine environments (>26 ppt), and its cultivation in China relies on raft culture and bottom-sowing culture [3,4]. In recent years, an industrial aquaculture system has been developed in China, which is characterized by significantly reduced water consumption and highly controllable water quality. This technological advancement enables sea urchin cultivation in inland areas with salt lakes or underground brine, provided that the salinity and essential elemental composition of the aquaculture water are precisely regulated to meet the species-specific physiological requirements.

Calcium, magnesium, and potassium ions are the principal cations in natural seawater, aside from sodium ions [5]. Inland salt lakes or underground brines frequently contain elevated concentrations of calcium, magnesium, and potassium ions [6,7]. For instance, the underground brines in the Qaidam Basin of Qinghai Province, China, as well as saline lake waters such as those in Qarhan Salt Lake, contain calcium, magnesium, and potassium ions at concentrations far higher than those found in seawater [8,9]. After dilution and adjustment, this source water shows potential for use in marine aquaculture operations. The appropriate ion concentration is crucial for aquaculture. High concentrations of calcium, magnesium, and potassium ions are often toxic to aquatic animals [10,11]. Their intake significantly impacts the growth and physiological functions of aquatic species. The mineral requirements and tolerance of aquatic animals vary substantially among species [12,13,14]. Currently, research on the effects of high concentrations of calcium, magnesium, and potassium ions on the survival and growth of sea urchins is unreported. Clarifying the impact of these elevated ions on sea urchins will help provide a favourable aquaculture water environment for them. Especially in regions where artificial seawater needs to be prepared using underground brine or salt-lake water with high concentrations of calcium, magnesium, and potassium ions, it is essential to clarify the impact of these ions on sea urchin aquaculture. Only by doing so can we determine whether further adjustments to calcium, magnesium, and potassium levels are necessary after salinity has been regulated to the appropriate range.

In the context of culturing sea urchins using artificial seawater prepared from high-salinity brines/salt-lake waters with elevated calcium, magnesium, and potassium ion concentrations, this study assessed the species’ tolerance to 1.5× and 2× natural seawater ion levels, systematically evaluating their impacts on survival, growth, and key physiological indices, thus providing technical parameters for aquaculture practices utilizing high-ion underground brine or salt-lake water.

## 2. Materials and Methods

### 2.1. Experimental Material

A total of 105 individuals (4–5 cm in test diameter) of *S. intermedius* were obtained from the Key Laboratory of Mariculture and Stock Enhancement in North China’s Sea, Ministry of Agriculture and Rural Affairs, Dalian Ocean University. Experimental seawater with elevated calcium, magnesium, or potassium ion concentrations was prepared by supplementing natural seawater with precise quantities of calcium chloride (CaCl_2_), magnesium chloride hexahydrate (MgCl_2_·6H_2_O), or potassium chloride (KCl) according to experimental design specifications. All chemical reagents used were of analytical grade, procured from Tianjin Kemiou Chemical Reagent Co., Ltd., Tianjin, China.

### 2.2. Experimental Design

The concentrations of calcium, magnesium, and potassium ions in normal seawater were determined based on the actual composition of local seawater in Dalian, with reference to the concentration ranges of major ions in oceanic water [15]. These concentrations were calculated at a salinity of 30.5 as follows: calcium ion = 366.5 mg/L, magnesium ion = 1151 mg/L, and potassium ion = 355 mg/L.

The experiment established 2 calcium ion levels, which were 1.5× and 2.0× the normal seawater concentration (actual concentrations of 550 mg/L and 733 mg/L, respectively); 2 magnesium ion levels, which were 1.5× and 2.0× the normal seawater concentration (actual concentration of 1727 mg/L and 2302 mg/L, respectively); and 2 potassium ion levels, which were 1.5× and 2.0× the normal seawater concentration (actual concentrations of 533 mg/L and 710 mg/L, respectively). In addition, normal seawater was used as the control group. Thus, 7 types of seawater were obtained for the experiment. Each treatment had 3 replicate groups, totalling 21 experimental units. The experiment used 21 buckets (10 L), filled with 8 L of seawater and stocked with 5 sea urchins per bucket. The sea urchins were acclimated until mortality ceased before experimentation. The experiment lasted for 45 days. Throughout the experimental period, the mean water temperature was maintained at 18.59 ± 1.02 °C and pH at 7.91 ± 0.03. The sea urchins were fed kelp (*Saccharina japonica*) to satiation. Water changes were performed every 3 days, with each experimental tank’s water replacement completed within 2 min and a temperature differential maintained at <0.5 °C to minimize the stress induced by the procedure.

### 2.3. Data Measurement

#### 2.3.1. Survival Rate

At the conclusion of the experiment, survival rates were calculated as follows:

Survival Rate (%) = 100 × (N_f_/N_i_)

where N_i_ and N_f_ represent the initial and final numbers of sea urchins, respectively.

#### 2.3.2. Growth

SGR: Body weights of all experimental sea urchins were measured at 15-day intervals. SGR was calculated for each measurement period using the following formula:

SGR (%/d) = 100 × (ln W_d_ − ln W_i_)/t

where W_d_ and W_i_ denote the final and initial mean body weight per replicate group at each stage and t represents the duration of the corresponding stage.

FCR: The weight of fresh kelp was measured during each feeding event, while the weight of rotten kelp was measured every 3 days. Total feed intake was determined at the experiment’s termination. The FCR was calculated as follows:

FCR = F/(W_t_ − W_0_) (1)

where W_0_ and W_t_ correspond to the initial and final mean body weights, respectively, and F indicates the total weight of feed consumed during the experimental period.

#### 2.3.3. Gonad Traits

At experimental termination, 3 sea urchins per tank were randomly selected and dissected in a tray to extract gonad tissue. Gonad wet weight (precision: 0.01 g) was recorded, and the gonad index (GI) was calculated as follows:

GI (%) = 100 × W_g_/W_f_ (2)

where W_g_ represents the gonad wet weight and W_f_ denotes the final body weight.

The gonad colour values L*, a*, and b* (CIELAB) were measured using a spectrophotometer, CM-2600d (Konica Minolta Sensing, Inc., Tokyo, Japan), and each sample was measured 3 times to obtain a mean value. The colour difference of each measured sea urchin against the ideal colour value (light orange-yellow: L* = 68.9, a* = 28.7, b* = 60.4; light yellow: L* = 74.6, a* = 28.7, and b* = 66.1) [16] was calculated according to the formula of CIEDE2000 [17,18]. A smaller ΔE value indicate closer chromatic similarity to the standard.

#### 2.3.4. Determination of Digestive Enzyme and Na^+^/K^+^-ATPase Activities

The sea urchins were dissected in trays to isolate intestines and tube feet (tube feet constitute one of the principal components of the echinoderm radial canal system, serving as a characteristic organ unique to this phylum [2]), which were immediately stored at −80 °C. For analysis, tissue samples were precisely weighed and homogenized in 10 volumes (*w*/*v* = 1:10, g/mL) of ice-cold physiological saline using an automated tissue homogenizer (CBFSTPRP-32, Shanghai, China). The homogenates were centrifuged at 2500 r/min for 10 min at 4 °C, and supernatants were collected for subsequent analyses. Total protein content was determined via the Coomassie Brilliant Blue method using commercial kits (Jiancheng Bioengineering Institute, Nanjing, China). The intestinal samples were used for the measurement of pepsin, amylase, and lipase, while the tube foot samples were used for the measurement of Na^+^/K^+^-ATPase. All enzymatic determinations were performed according to the manufacturer’s protocols (Jiancheng Bioengineering Institute, Nanjing, China) using a microplate reader (Epoch™, BioTek Instruments, Inc., Winooski, VT, USA).

#### 2.3.5. Determination of Immune Enzyme Activity

Coelomic fluid was aspirated from the peristomial region of sea urchins using a 1 mL disposable syringe and transferred to 1.5 mL sterile centrifuge tubes. Cellular samples were centrifuged at 3000 r/min for 10 min, with all samples stored at −80 °C. Total protein content was determined via the Bicinchoninic Acid Assay (BCA). Immune-related enzymatic activities were measured using commercial kits (Jiancheng Bioengineering Institute, Nanjing, China): acid phosphatase (ACP), lysozyme, and superoxide dismutase (SOD).

### 2.4. Statistical Analysis

Experimental data are presented as mean ± S.D. Data for the calcium and potassium ion groups were analysed by one-way ANOVA using SPSS 26.0 (IBM Corporation, Armonk, NY, USA) software, with post hoc LSD tests for multiple comparisons. Data for the magnesium ion group were analysed using independent sample *t*-tests. The significance level was set at 0.05.

## 3. Results

### 3.1. Survival Rate of S. intermedius Under Different Calcium, Magnesium, and Potassium Ion Concentrations

In the 2302 mg/L magnesium ion group, all experimental sea urchins died within 2 days. After 45 days of culturing, the survival rate was 86.7% in the 733 mg/L calcium ion group and 73.3% in the 1727 mg/L magnesium ion group. Survival rates were 100% in the 550 mg/L calcium ion group, the 533 mg/L potassium ion group, the 710 mg/L potassium ion group, and the control group (as shown in Table 1).

**Figure 1:**
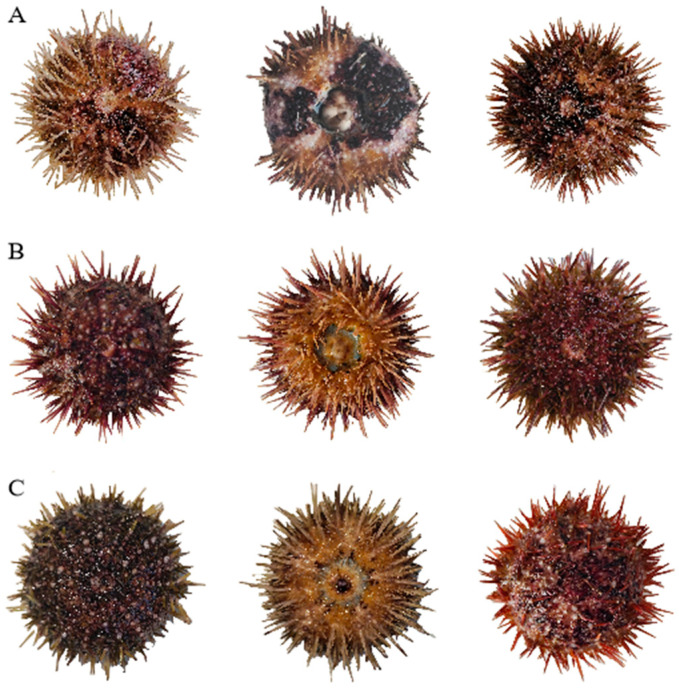
Morphological comparison of *S. intermedius* specimens lethally affected by elevated concentrations of (**A**) calcium ions, (**B**) magnesium ions, and (**C**) potassium ions.

The appearance of sea urchins lethally affected by high concentrations of calcium, magnesium, and potassium ions is shown in Figure 1, where Figure 1A, 1B, and 1C represent sea urchins killed by high concentrations of calcium, magnesium, and potassium ions, respectively.

### 3.2. SGR of S. intermedius Under Different Calcium, Magnesium, and Potassium Ion Concentrations

**Figure 2:**
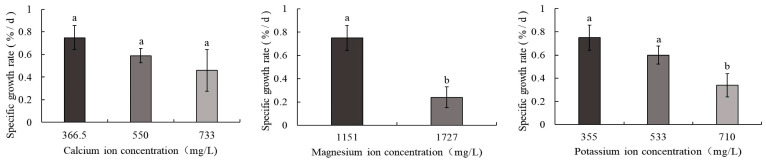
Comparison of SGR of body weight in *S. intermedius* among different experimental groups. Note: Different lowercase letters denote statistically significant differences among groups (*p* < 0.05).

As shown in Figure 2, the control group displayed the highest SGR of body weight at 0.75%/d, whereas the 1727 mg/L magnesium ion treatment group exhibited the lowest SGR (0.24%/d). A consistent decline in SGR was observed in *S. intermedius* with increasing calcium, magnesium, and potassium ion concentrations. Significant differences (*p* < 0.05) in SGR were detected between the control group and both the 1727 mg/L magnesium ion and 710 mg/L potassium ion treatment groups. In contrast, no statistically significant differences were observed between the control group and the 550 mg/L calcium ion, 733 mg/L calcium ion, or 533 mg/L potassium ion treatment groups.

### 3.3. FCR of S. intermedius Under Different Calcium, Magnesium, and Potassium Ion Concentrations

**Figure 3:**
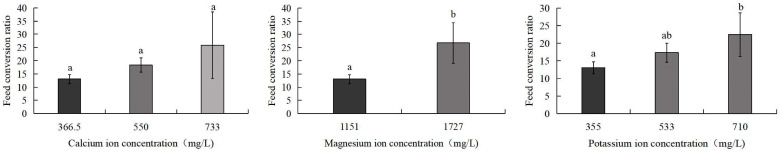
Comparison of FCR of *S. intermedius* among different experimental groups. Different lowercase letters denote statistically significant differences among groups (*p* < 0.05).

As shown in Figure 3, the FCR of *S. intermedius* exhibited an increasing trend with elevated calcium, magnesium, and potassium ion concentrations. The control group demonstrated the lowest FCR (13.0), which was significantly lower than the 1727 mg/L magnesium ion group (26.8) and 710 mg/L potassium ion group (22.5) (*p* < 0.05). No significant differences were observed between the control group and the 550 mg/L calcium ion, 733 mg/L calcium ion, or 533 mg/L potassium ion treatment groups.

### 3.4. GI of S. intermedius Under Different Calcium, Magnesium, and Potassium Ion Concentrations

**Figure 4:**
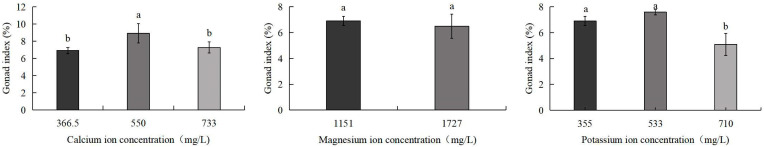
Comparison of GI of *S. intermedius* among different experimental groups. Different lowercase letters denote statistically significant differences among groups (*p* < 0.05).

Figure 4 demonstrates that the GI of *S. intermedius* exhibited an initial increase followed by a decline with rising calcium ion and potassium ion concentrations. The 550 mg/L calcium ion group exhibited the highest GI (8.9%), significantly exceeding both the control group (6.9%) and the 733 mg/L calcium ion group (7.3%) (*p* < 0.05). While the 533 mg/L potassium ion group showed a higher GI than the control, this difference lacked statistical significance. Conversely, the 710 mg/L potassium ion group displayed the lowest GI (5.1%), significantly lower than both the control and 533 mg/L K^+^ groups (*p* < 0.05). The gonad index of sea urchins in the 1727 mg/L magnesium ion group showed no significant difference compared to the control group.

### 3.5. Gonad Colour Variation of S. intermedius Under Different Calcium, Magnesium, and Potassium Ion Concentrations

**Figure 5:**
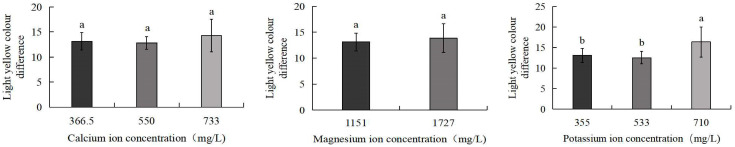
Comparison of light yellow colour difference of *S. intermedius* among different experimental groups. Different lowercase letters denote statistically significant differences among groups (*p* < 0.05).

**Figure 6:**
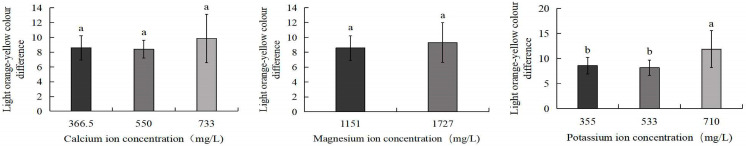
Comparison of light orange-yellow colour difference of *S. intermedius* among different experimental groups. Different lowercase letters denote statistically significant differences among groups (*p* < 0.05).

As shown in Figure 5, the 710 mg/L potassium ion group exhibited the greatest colour difference from light yellow (16.3), which was significantly higher than the control group (13.1) and the 533 mg/L potassium ion group (12.5) (*p* < 0.05). No significant differences were observed between the control group and the 550 mg/L calcium ion, 733 mg/L calcium ion, 1727 mg/L magnesium ion, or 533 mg/L potassium ion treatment groups. Figure 6 further demonstrates that the 710 mg/L potassium ion group displayed the largest colour difference from light orange-yellow (11.8), significantly exceeding the control (8.6) and 533 mg/L potassium ion groups (8.1) (*p* < 0.05). Similarly, no statistically significant differences were detected between the control group and the 550 mg/L calcium ion, 733 mg/L calcium ion, 1727 mg/L magnesium ion, or 533 mg/L potassium ion treatment groups.

### 3.6. Effects of Calcium, Magnesium, and Potassium Ion Concentrations on Digestive Enzyme Activities in S. intermedius

#### 3.6.1. Pepsin

**Figure 7:**
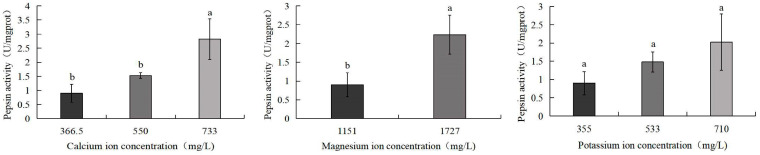
Comparison of pepsin activity of *S. intermedius* among different experimental groups. Different lowercase letters denote statistically significant differences among groups (*p* < 0.05).

Figure 7 indicates that pepsin activity in *S. intermedius* increased with rising calcium, magnesium, and potassium ion concentrations. The 733 mg/L calcium ion group showed the highest pepsin activity (2.8 U/mg), significantly surpassing both the control group (0.9 U/mg) and the 550 mg/L calcium ion group (1.5 U/mg) (*p* < 0.05). Similarly, the 1727 mg/L magnesium ion group exhibited significantly elevated pepsin activity compared to the control (*p* < 0.05). No significant differences were observed between the control group and the 533 mg/L potassium ion or 710 mg/L potassium ion treatment groups.

#### 3.6.2. Amylase

**Figure 8:**
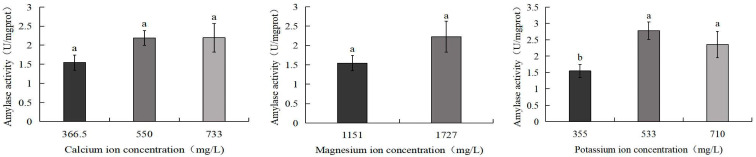
Comparison of amylase activity of *S. intermedius* among different experimental groups. Different lowercase letters denote statistically significant differences among groups (*p* < 0.05).

Figure 8 demonstrates that the amylase activity of *S. intermedius* exhibited an initial increase followed by a decline with rising potassium ion concentrations. The 533 mg/L potassium ion group displayed the highest amylase activity (2.8 U/mg), significantly exceeding the control group (1.5 U/mg) (*p* < 0.05). Similarly, the 710 mg/L potassium ion group also showed significantly elevated amylase activity compared to the control (*p* < 0.05). No significant differences were observed between the control group and the 550 mg/L calcium ion, 733 mg/L calcium ion, or 1727 mg/L magnesium ion treatment groups.

#### 3.6.3. Lipase

**Figure 9:**
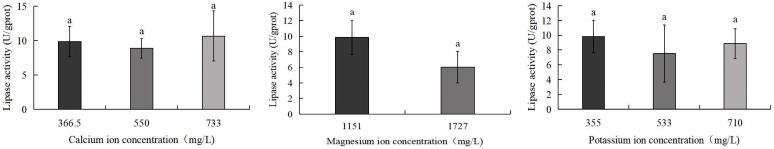
Comparison of lipase activity of *S. intermedius* among different experimental groups. Different lowercase letters denote statistically significant differences among groups (*p* < 0.05).

As illustrated in Figure 9, the 733 mg/L calcium ion group exhibited the highest lipase activity (10.6 U/gprot), while the 1727 mg/L magnesium ion group showed the lowest activity (6.0 U/gprot). However, no statistically significant differences were detected among any experimental groups.

### 3.7. Effects of Calcium, Magnesium, and Potassium Ion Concentrations on Immune Enzyme Activities in S. intermedius

#### 3.7.1. ACP

**Figure 10:**
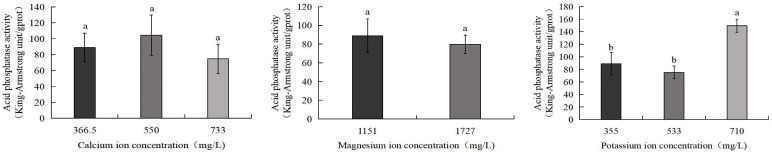
Comparison of ACP activity of *S. intermedius* among different experimental groups. Different lowercase letters denote statistically significant differences among groups (*p* < 0.05).

As shown in Figure 10, the 710 mg/L potassium ion group exhibited the highest ACP activity (149.4 King’s units/gprot), which was significantly higher than both the control group (88.9 King’s units/gprot) and the 533 mg/L potassium ion group (75.1 King’s units/gprot) (*p* < 0.05). No statistically significant differences were observed between the control group and the 550 mg/L calcium ion, 733 mg/L calcium ion, or 1727 mg/L magnesium ion treatment groups.

#### 3.7.2. Lysozyme

**Figure 11:**
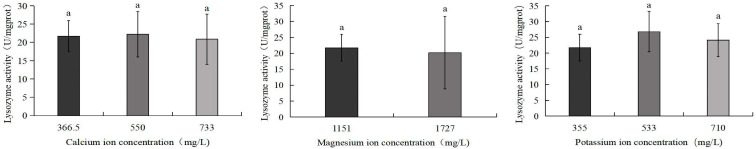
Comparison of lysozyme activity of *S. intermedius* among different experimental groups. Different lowercase letters denote statistically significant differences among groups (*p* < 0.05).

As illustrated in Figure 11, the 533 mg/L potassium ion group exhibited the highest lysozyme activity (26.8 U/mg), whereas the 1727 mg/L magnesium ion group displayed the lowest activity (20.2 U/mg). However, no statistically significant differences were detected among any experimental groups.

#### 3.7.3. SOD

**Figure 12:**
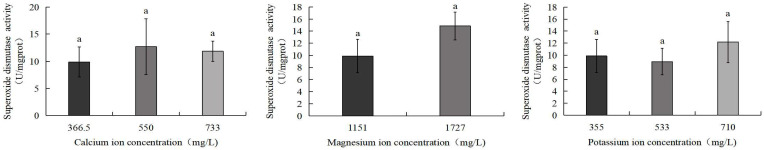
Comparison of SOD activity of *S. intermedius* among different experimental groups. Different lowercase letters denote statistically significant differences among groups (*p* < 0.05).

Figure 12 demonstrates that the 1727 mg/L magnesium ion group showed the highest SOD activity (14.9 U/mg), while the 533 mg/L potassium ion group exhibited the lowest activity (8.9 U/mg). Similarly, no statistically significant differences were detected among any experimental groups.

### 3.8. Effects of Calcium, Magnesium, and Potassium Ion Concentrations on Na^+^/K^+^-ATPase Activity in S. intermedius

**Figure 13:**
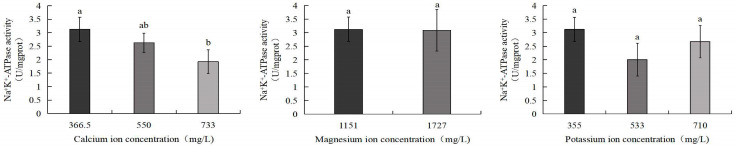
Comparison of Na^+^/K^+^-ATPase activity of *S. intermedius* among different experimental groups. Different lowercase letters denote statistically significant differences among groups (*p* < 0.05).

As shown in Figure 13, Na^+^/K^+^-ATPase activity in *S. intermedius* exhibited a declining trend with increasing calcium ion concentrations. The control group displayed the highest Na^+^/K^+^-ATPase activity (3.1 U/mg), which was significantly higher than the 733 mg/L calcium ion group (1.9 U/mg) (*p* < 0.05). No significant differences were observed between the control group and the 550 mg/L calcium ion, 1727 mg/L magnesium ion, 533 mg/L potassium ion, or 710 mg/L potassium ion treatment groups.

## 4. Discussion

### 4.1. Tolerance of S. intermedius to High Concentrations of Calcium, Magnesium, and Potassium Ions

In this experiment, the results revealed that *S. intermedius* exhibited the strongest tolerance to potassium ions, achieving 100% survival after 45 days under the 2× natural seawater K^+^ concentration (710 mg/L). While calcium tolerance was also robust, survival under the 2× calcium ion concentration (733 mg/L) reached 86.7%, although lower than the 100% survival observed in the 2× potassium ion group. In contrast, magnesium proved most toxic: all individuals died within 2 days under the 2× magnesium ion concentration (2302 mg/L), and even at the 1.5× magnesium ion concentration (1727 mg/L), 26.7% mortality occurred, whereas no deaths were observed in the 1.5× calcium ion or potassium ion groups. These findings establish a tolerance hierarchy of potassium ion > calcium ion > magnesium ion for *S. intermedius*. Practically, this implies that if aquaculture water sources contain high magnesium, concentrations should be reduced to natural seawater levels to ensure survival. Conversely, potassium concentrations below 710 mg/L and calcium below 550 mg/L may remain unadjusted without compromising survival rates.

### 4.2. Influences of Calcium, Magnesium, and Potassium Ion Concentrations on SGR, FCR, GI, and Gonad Colour

Previous studies have demonstrated that most aquatic species can directly absorb calcium from their ambient environment to meet physiological demands [19,20,21,22], with absorption efficiency dependent on seawater calcium ion concentrations. Calcium plays critical roles in the structure of hard tissues, blood coagulation, muscular contraction, osmoregulation, neural transmission, and cell signalling [23,24,25,26]. Acting as a second messenger intracellularly [27], calcium is essential for physiological functions, and both deficiency and excess can disrupt cellular functions [28]. In this study, as calcium ion concentration increases, the SGR of body weight exhibits a declining trend, while the FCR showed an increasing trend. However, neither of these changes reached statistical significance. This indicates that the high calcium ion concentrations employed in this experiment have not yet exerted significant adverse effects on the physiological functions of *S. intermedius* related to feed conversion and somatic growth. The 550 mg/L calcium ion treatment group exhibited a significant increase in GI, suggesting that adequate calcium ion levels enhance gonadal development in *S. intermedius*. The current research on gonadal development in aquaculture predominantly focuses on artificial compound feed. Cuesta-Gomez et al. [29] reported that feed with soybean lecithin and fish oil promotes gonadal development in the purple sea urchin (*Strongylocentrotus purpuratus*). However, calcium utilization in feeds is influenced by multiple factors, including calcium content, calcium sources, interactions with other feed components, and the calcium utilization capacity of aquatic animals [30]. Aquatic animals cannot fully utilize feed calcium [31], highlighting the importance of seawater calcium ion supplementation for gonadal development. Notably, while no significant difference in SGR was observed between the control and 550 mg/L calcium ion groups, the latter showed a marked elevation in the GI. These findings suggest that supplementation of calcium ions in *S. intermedius* could effectively enhance the GI. The high calcium ion concentrations used in this study did not exert adverse effects on gonad colour, suggesting that calcium ions may not be directly involved in the physiological process of gonadal coloration.

Magnesium, a vital cation in biological systems participating in skeletal and dental composition, catalyses and activates over 300 enzymatic systems and profoundly influences energy metabolism, protein metabolism, sugar metabolism, and lipid metabolism in aquatic animals [32]. In this study, the control group under normal ionic conditions exhibited the highest SGR, indicating that balanced ionic environments are most favourable for the growth of sea urchins. However, elevating seawater magnesium ion concentrations to 1727 mg/L significantly suppressed growth and increased the FCR, demonstrating that high magnesium concentrations can disrupt the ion balance of sea urchins, ultimately limiting growth and even mortality. Excessive magnesium intake has been reported in fish, where excessive dietary magnesium increases excretory burdens, disturbs ion balance, impairs the absorption of other elements, and adversely affects hematopoietic functions [33,34]. Han et al. found that a dietary magnesium level of 2900 mg/kg had negative effects on the survival rate and growth efficiency of crucian carp (*Carassius auratus gibelio*) [35].

Potassium ions play a crucial role in maintaining osmolality and the humoral acid–base balance [36,37], preserving cellular morphology and sustaining the normal performance of muscles and nerves. Furthermore, potassium ions synergistically interact with mineral elements including calcium ions, magnesium ions, and sodium ions to participate in critical biological processes such as carbohydrate metabolism [38,39]. However, existing studies indicate that excessive potassium ion supplementation is unnecessary in seawater or feed. For example, under salinity 30 conditions, *Litopenaeus vannamei* exhibits limited capacity to utilize dietary potassium, and potassium supplementation in feed inhibits shrimp growth when ambient potassium is sufficient [40]. Research by Sakamoto et al. [41] also concluded that additional potassium supplementation is unnecessary in diets for red sea bream (*Pagrus major*) due to naturally high potassium concentrations in seawater. In the present experiment, when the seawater potassium concentration reached 710 mg/L, the specific growth rate of sea urchins was suppressed, the gonad index significantly decreased (*p* < 0.05), and the FCR increased. Furthermore, the gonad colour differences in this concentration group were significantly higher than those in the control group. These results align with previous studies, further confirming that excessive potassium ion addition to seawater is unnecessary.

### 4.3. Influences of Calcium, Magnesium, and Potassium Ion Concentrations on Digestive and Immune Enzyme Activities

Digestive enzyme activity reflects nutrient utilization efficiency in sea urchins, with enzymatic levels critically impacting growth rates [42,43]. Our results demonstrated significant intergroup differences in amylase and pepsin activities (*p* < 0.05). Amylase activity in the 533 mg/L potassium ion and 710 mg/L potassium ion groups was significantly higher than in controls, indicating that adequate potassium ion concentrations enhance amylase activity in *S. intermedius*. Calcium ion concentration exerted a pronounced effect on pepsin activity (*p* < 0.05), showing an increasing trend with rising ion concentrations. Notably, the 1727 mg/L magnesium ion group also had significantly elevated pepsin activity, likely associated with the roles of calcium ions and magnesium ions in pepsin activity. Studies on kuruma prawn (*Marsupenaeus japonicus*) by Galgani et al. demonstrated that calcium ions and magnesium ions activate trypsin at a concentration of 5 × 10^−3^ mol/L [44]. Research on swimming crabs (*Portunus trituberculatus*) showed that calcium ions activate protease [45]. Although the elevated concentrations of the three ions significantly enhanced digestive enzyme activity, they did not promote somatic growth in sea urchins. This discrepancy may be attributed to the fact that while the enhanced digestive capacity improved food breakdown, the physiological functions related to nutrient absorption, transport, and metabolic conversion were adversely affected by the high ion concentrations, which requires further investigation.

Invertebrates lack specific immune systems and primarily rely on humoral immunity mediated by non-specific immune factors to combat pathogen invasion [46]. Crucially, ACP participates in multiple immune processes, such as pathogen recognition, phagocytosis, and microbial elimination [47]. Metal ions exert divergent effects on enzyme activity, with even those possessing modulatory functions demonstrating substantially varying impacts on enzymatic structure and function [48]. Metal ions may alter enzyme conformation and activity through binding to active sites of enzymes [49]. In the present study, calcium ions and magnesium ions exhibited minimal effects on ACP activity; 710 mg/L of potassium ions demonstrated moderate ACP activation. Gao et al. reported negligible potassium ion effects on crucian carp (*Carassius auratus*) ACP, while calcium ions/magnesium ions showed concentration-dependent inhibitory effects [50]. The effects of different concentrations of calcium, magnesium, and potassium ions on the activities of other immune-related enzymes in *S. intermedius*, such as lysozyme and SOD, were not significant. This may be because the activities of these enzymes are more strongly influenced by other environmental factors than by ion concentrations.

Na^+^/K^+^-ATPase, a critical component of the sodium–potassium pump, participates in the active transmembrane transport of sodium ions and potassium ions while maintaining physiological ion gradients across cellular membranes. Its activity serves as a key for osmoregulatory competence in aquatic organisms [51,52]. Calcium ions are a fundamental constituent of natural seawater and play a significant role in the osmoregulation of aquatic organisms. In this study, increasing calcium ion concentrations induced a progressive decline in Na^+^/K^+^-ATPase activity in *S. intermedius*, potentially attributable to calcium ion–potassium ion ionic antagonism. Giese (1986) demonstrated that isosmotic CaCl_2_ solutions reduced water permeability in kelp, whereas KCl solutions enhanced membrane permeability, establishing their functional antagonism [53]. Dennis (1982) further elucidated that calcium ions modulate membrane permeability to potassium ions, with elevated ambient calcium ion concentrations suppressing potassium ion influx and consequently inhibiting Na^+^/K^+^-ATPase activity [54]. This inhibition may disrupt intracellular–extracellular ion gradients, impairing osmoregulatory capacity. The effects of salinity changes and low potassium ion concentrations on Na^+^/K^+^-ATPase activity have been previously reported. American eels (*Anguilla rostrata*), Japanese eels (*A. japonica*), and European eels (*A. anguilla*) exhibit a salinity-dependent upregulation of branchial Na^+^/K^+^-ATPase during marine migration [55,56]. Potassium ion deficiency or low potassium ion concentrations in the aquatic environment can inhibit the activity of Na^+^/K^+^-ATPase [57]. A study by Perez-Velazquez et al. showed that when Pacific white shrimp were cultured at temperatures of 20 °C and 24 °C, insufficient potassium ion levels or excessively high Na^+^/K^+^ ratios negatively impacted the growth and survival of the shrimp [58]. In this experiment, the salinity did not change, and the increase in potassium ion concentration may not lead to the imbalance of intracellular–extracellular ion gradients and had no significant effect on Na^+^/K^+^-ATPase activity.

## 5. Conclusions

Based on the aforementioned findings, we conclude that artificial seawater formulated with 550 mg/L calcium ions or 533 mg/L potassium ions is biologically acceptable for *S. intermedius* aquaculture. This concentration range exerts no statistically significant adverse effects on growth performance, gonad colour, or immune enzyme activities, while simultaneously enhancing the gonad index and digestive enzyme activity to a beneficial extent. *S. intermedius* exhibits sensitivity to magnesium ion concentrations; when formulating artificial seawater, it is advisable to restrict magnesium ions to a narrow optimal range to ensure survival and health.

## Figures and Tables

**Table 1:** Effects of long-term stress from high concentrations of calcium, magnesium, and potassium ions on the survival rate of *S. intermedius*.

Groups	Concentrations (mg/L)	Items	
		Survival Counts (Mean ± S.D.)	Survival Rates (Mean ± S.D.)
calcium ion	550	15 ± 0	100% ± 0.0%
	733	13 ± 0.5	86.7% ± 11.6%
magnesium ion	1727	11 ± 0.5	73.3% ± 11.6% *
	2302	0 ± 0	0% ± 0.0% *
potassium ion	533	15 ± 0	100% ± 0.0%
	710	15 ± 0	100% ± 0.0%
control	calcium ion 366.5, magnesium ion 1151, and potassium ion 355	15 ± 0	100% ± 0.0%

* Indicates a significant difference from the control group (*p* < 0.05).

## Data Availability

The original contributions presented in the study are included in the article; further inquiries can be directed to the corresponding authors.
